# Gap in Willingness and Access to Video Visit Use Among Older High-risk Veterans: Cross-sectional Study

**DOI:** 10.2196/32570

**Published:** 2022-04-08

**Authors:** Stuti Dang, Kiranmayee Muralidhar, Shirley Li, Fei Tang, Michael Mintzer, Jorge Ruiz, Willy Marcos Valencia

**Affiliations:** 1 Geriatric Research, Education and Clinical Center Miami Veterans Affairs Healthcare System Miami, FL United States; 2 Division of Geriatrics and Palliative Care Miller School of Medicine University of Miami Miami, FL United States; 3 The Elizabeth Dole Center of Excellence for Veteran and Caregiver Research Miami, FL United States; 4 Department of Epidemiology Miller School of Medicine University of Miami Miami, FL United States; 5 Miller School of Medicine University of Miami Miami, FL United States; 6 Division of Endocrinology, Diabetes and Metabolic Diseases Department of Medicine Medical University of South Carolina Charleston, SC United States

**Keywords:** high-risk veterans, older adults, telemedicine, video visits, health disparities, Area Deprivation Index, mobile phone

## Abstract

**Background:**

The recent shift to video care has exacerbated disparities in health care access, especially among *high-need, high-risk (HNHR)* adults. Developing data-driven approaches to improve access to care necessitates a deeper understanding of HNHR adults’ attitudes toward telemedicine and technology access.

**Objective:**

This study aims to identify the willingness, access, and ability of HNHR veterans to use telemedicine for health care.

**Methods:**

WWe designed a questionnaire conducted via mail or telephone or in person. Among HNHR veterans who were identified using predictive modeling with national Veterans Affairs data, we assessed willingness to use video visits for health care, access to necessary equipment, and comfort with using technology. We evaluated physical health, including frailty, physical function, performance of activities of daily living (ADL) and instrumental ADL (IADL); mental health; and social needs, including Area Deprivation Index, transportation, social support, and social isolation.

**Results:**

The average age of the 602 HNHR veteran respondents was 70.6 (SD 9.2; range 39-100) years; 99.7% (600/602) of the respondents were male, 61% (367/602) were White, 36% (217/602) were African American, 17.3% (104/602) were Hispanic, 31.2% (188/602) held at least an associate degree, and 48.2% (290/602) were confident filling medical forms. Of the 602 respondents, 327 (54.3%) reported willingness for video visits, whereas 275 (45.7%) were unwilling. Willing veterans were younger (*P*<.001) and more likely to have an associate degree (*P*=.002), be health literate (*P*<.001), live in socioeconomically advantaged neighborhoods (*P*=.048), be independent in IADLs (*P*=.02), and be in better physical health (*P*=.04). A higher number of those willing were able to use the internet and email (*P*<.001). Of the willing veterans, 75.8% (248/327) had a video-capable device. Those with video-capable technology were younger (*P*=.004), had higher health literacy (*P*=.01), were less likely to be African American (*P*=.007), were more independent in ADLs (*P*=.005) and IADLs (*P*=.04), and were more adept at using the internet and email than those without the needed technology (*P*<.001). Age, confidence in filling forms, general health, and internet use were significantly associated with willingness to use video visits.

**Conclusions:**

Approximately half of the HNHR respondents were unwilling for video visits and a quarter of those willing lacked requisite technology. The gap between those willing and without requisite technology is greater among older, less health literate, African American veterans; those with worse physical health; and those living in more socioeconomically disadvantaged neighborhoods. Our study highlights that HNHR veterans have complex needs, which risk being exacerbated by the video care shift. Although technology holds vast potential to improve health care access, certain vulnerable populations are less likely to engage, or have access to, technology. Therefore, targeted interventions are needed to address this inequity, especially among HNHR older adults.

## Introduction

### Background

The onset of the COVID-19 pandemic led to sudden and dramatic changes in the delivery of health care in the context of social distancing and lockdown decisions. Telemedicine has emerged as a solution to caring for patients who are medically complex during the pandemic [1]. Institutions have diverted resources toward purchasing necessary telemedicine equipment and expansion of technological infrastructure and hastily implemented telemedicine training sessions for providers [2,3]. Telemedicine reimbursement models also saw formula adjustments. For example, the Centers for Medicare and Medicaid Services (CMS) insurance models changed in March 2020 to reduce the costs of telemedicine [4], and the CMS issued waivers that allowed providers to care for patients remotely without financial penalties [5]. These factors have contributed to the accelerated implementation of telemedicine across health care systems [2,3].

The Veterans Affairs (VA) has been a leader in integrating the use of technology into health care. The implementation of telemedicine technologies and new programs at the VA has accelerated in recent years to expand access to more veterans. Since 2018, the VA’s *Anywhere to Anywhere* initiative expanded the scope of telehealth so that care can be delivered via telehealth across state borders and even in the veterans’ homes [6]. During the COVID-19 pandemic, similar to other health care systems, the VA moved rapidly to leverage its telemedicine capabilities to provide needed care to veterans at home [2,7]. A major pivot by the VA during the COVID-19 pandemic was the rapid adoption and use of the VA’s telemedicine platform, Veteran Video Connect (VVC), which allowed most visits to be done via telemedicine at home. VVC is a videoconferencing application for veterans and their providers. It securely connects veterans to their health care team from any internet-enabled computer, tablet, or mobile device. In the face of this public health emergency, the VA also suspended previous Health Insurance Portability and Accountability Act compliance requirements to allow providers to connect with patients on non–public-facing technology if VVC was not working or at overcapacity [8].

Nevertheless, despite the rapid pivot to telemedicine, there have been valid concerns regarding patient-level challenges to wider implementation and integration of technology into health care. Using 2018 data from the National Health and Aging Trends Study of community-dwelling adults, Lam et al [9] estimated that approximately one-third of the older adults in the United States were not ready for video visits, which is largely attributed to inexperience with technology. Individuals who face barriers to accessing care in person are also likely the same individuals who face challenges accessing telemedicine and include those who are older and minority; have lower educational attainment, lower income, and self-reported poor health status [9-11]; and live in rural areas [9,12-14]. Therefore, disparities in health care access risk exacerbation by the ongoing shift to adopt telemedicine [9,11], especially among the highest risk patients with the most complex clinical scenarios [9,12].

### Objective

To develop data-driven approaches and understand how best to deploy telemedicine to increase access to care for older adults who are complex and frail, it would be beneficial to form a deeper understanding of their attitude toward using video visits for receiving health care. Using a population health approach, the VA identifies a subgroup of veterans called *high-need, high-risk (HNHR)* veterans, who represent the VA population that would qualify for Medicare’s demonstration of home-based primary care (HBPC; ie, independence at home) [15]. The primary aim of this study is to evaluate HNHR older veterans’ willingness, access, and ability to use video visits for health care purposes. Our secondary aim is to characterize the willingness for telemedicine in the context of their physical, emotional, and social determinants. Our hypothesis is that among HNHR older adults, the access and ability to use video visits would be lower than that shown previously among community-dwelling adults [9].

Ultimately, this paper seeks to add to the ongoing efforts to provide actionable data that may help health care systems leverage telemedicine as a means of increasing access to health care. We can expect the increased reliance on telemedicine to be sustained, and increasing our understanding of the factors contributing to digital disparities will help identify targeted interventions to address the identified challenges to telemedicine for HNHR patients, who are also the patients most likely in need of support.

## Methods

### Overview

This cross-sectional observational study was part of a larger quality improvement study to better define the needs of HNHR veterans in the Miami VA Healthcare System. Here, we analyzed the willingness, technology access, and ability to use video visits in the HNHR veteran group.

### Study Population

The VA Geriatrics and Extended Care Data Analysis Center uses population health VA data to identify HNHR veterans who are medically complex and functionally impaired and at the highest risk for hospitalization and long-term institutionalization and, therefore, eligible for HBPC. The criteria for the Geriatrics and Extended Care Data Analysis Center HNHR designation include hospitalization in the prior 12 months and medical complexity measures that include the 13-condition JEN Frailty Index (JFI) [16] score ≥6, suggesting dependency in ≥2 activities of daily living (ADL), and NOSOS (VA version of the CMS measure to project cost). Patients were excluded if they had end-stage renal disease; were enrolled in HBPC or medical foster home; had received hospice, palliative care, or nursing home care in the past 12 months; or lived >60 minutes away from the closest VA primary care site as VA HBPC programs were less likely to be available at this distance [15].

Over a 1-year period that extended from October 2017 to September 2018, 2543 Miami VA Healthcare System veterans were listed as HNHR. Of those 2543 veterans, 1300 (51.12%) were randomly selected and sent a questionnaire via the US Postal Service. The mailings were sent in two waves: May 2018 and November 2018. The questionnaires were conducted by mail only once, with no reminders to improve the response rate. An additional group of 173 HNHR veterans scheduled for a geriatric frailty clinic appointment completed the questionnaire.

### Questionnaire Design and Variables

We designed a questionnaire to assess physical health, including frailty—with the Fatigue, Resistance, Ambulation, Illnesses, and Loss of Weight scale [17]—physical function, mobility, ADL [18], instrumental ADL (IADL) [19], and homebound status [20]; assess mental health using the Patient Health Questionnaire [21] for depression screening and perception of aging [22]; and assess social support, social isolation [23], and transportation. We assessed for willingness to use video visits for VA health care; among those willing to use video visits, we asked about access to the video-capable technology. Furthermore, we assessed the ability to use technology by asking about comfort in performing an internet search and using email. We also asked about My Health*e*Vet use and access and the desired mode of communication with VA. The used questions were either study specific, validated, or modified from validated questions. The details of the questionnaire are presented in Table 1. We have tried to segment and label our variables into those that relate to the level of the patient’s need for telehealth versus barriers and facilitators that we can do something about, although this distinction is somewhat arbitrary and case dependent, as only some of the factors are addressable some of the time. Physical and mental health characteristics may often relate to the level of patient need for telehealth but may also present a barrier, whereas the social and technology characteristics are the surrounding factors that act as facilitators or barriers, depending on the situation.

**Table 1:** Survey components.

Indicator		Source	Details
**Demographics**			
	Education	Study specific	Highest level of education completed
	Health literacy [24]	Question to identify patients with inadequate health literacy	Confidence filling medical forms; score ranged from 1 to 5, with a higher score indicating more confidence; a score of 5 was considered health literate
**Physical health (need or barrier)**			
	Frailty [17]	5-item FRAIL^a^ scale	The 5-item FRAIL scale includes fatigue, resistance, ambulation, illness, and weight loss. The final score ranges from 0 to 5 and represents frail (score 3-5), prefrail (score 1-2), and robust (score 0) health status. A score of 3 to 5 was considered a positive screen.
	General health [25]	Modified from the Stanford Chronic Disease Self-Management Program Questionnaire	Self-rated general health; scores ranged from 1 to 5, with a higher score indicating better self-rated general health
	Self-rated physical status	Self-rated physical status	Scores for self-rated physical status ranged from 1 to 10, with a higher score indicating better physical status
	Walking, falls, and exercise	Study specific	Issues with walking, stepping, and balance; assistive devices used; number of falls in the past year; barriers to exercise; pedometer use
	ADL^b^ [18]	Barthel index for ADL	Barthel ADL score (range 0-100), with a higher score indicating greater independence
	IADL^c^ [19]	Lawton score for IADL	Lawton IADL score (range 0-8), with a higher score indicating greater independence
	Homebound status [20]	Determining homebound status as part of a mobility questionnaire using validated questions from the National Health and Aging Trends Study	Individuals were categorized as homebound, semihomebound, and not homebound based on their responses to how often they left their home, how much help they had in leaving their home, and how much difficulty they had in leaving their home in the previous month, similar to the reference study.
**Mental health (need or barrier)**			
	Depression screen [21]	PHQ-2^d^	PHQ-2 scores ranged from 0 to 6; a score ≥3 is considered positive for the likelihood of depression
	Self-perception of aging [22]	Attitude Toward Own Aging subscale of the Philadelphia Geriatric Center Morale Scale	The 5-question scale (range 0-5) was treated as a binary variable. For the first (*feeling worse as I get older*) and third (*feeling useless as I get older*) questions on the scale, the responses *strongly disagree, disagree, somewhat disagree* were scored as 0, whereas the responses *somewhat agree, agree, strongly agree* were scored as 1. The responses to the second (*as much pep as last year*), fourth (*as happy as when I was younger*), and fifth (*things are better than I thought it would be*) questions were scored in a reverse manner. A higher score indicated a negative perception of aging.
**Social characteristics (facilitator or barrier)**			
	Social support	Study specific	Having a formal or informal caregiver; caregiver’s distance from home
	Social isolation [23]	Berkman–Syme Social Network Index	Scoring was performed as the following: married (no=0; yes=1), meeting and talking to close friends and relatives (<3 times a week=0; ≥3 times a week=1), participation in religious meetings or services (<4 times a year=0; ≥4 times a year=1), and attend meetings of the clubs or organizations (never or does not belong=0, all the responses=1). Scores were summed: 0 or 1 being the most isolated category, and 2, 3, or 4 formed the other 3 categories of increasing social integration.
	Transportation [26]	Questions assessing transportation barriers	Trouble with transportation, delayed physicians’ appointments because of transportation troubles, and travel time from home to their physician
**Technology (facilitator or barrier)**			
	Technology willingness, access, and ability	Study specific	Willingness to use video visits with VA^e^ providers; access to video-capable equipment among those willing to use video visits; ability to do an internet search and use email; My Health*e*Vet enrollment and use; preferred mode of contact

^a^FRAIL: Fatigue, Resistance, Ambulation, Illnesses, and Loss of Weight.

^b^ADL: activities of daily living.

^c^IADL: instrumental activities of daily living.

^d^PHQ-2: Patient Health Questionnaire-2.

^e^VA: Veterans Affairs.

Additional measures obtained from VA records included the Care Assessment Needs score (VA measure for hospitalization and mortality risk) [27] and the Hierarchical Condition Categories score [28]. We also obtained the Area Deprivation Index (ADI), an established measure of socioeconomic disadvantage at the census tract level, from the Neighborhood Atlas [29].

### Statistical Analysis

Descriptive characteristics were presented as frequency (percentage) for categorical variables and as mean (SD) for continuous variables. We compared the characteristics of respondents who were willing to use video visits with those who were not; among those willing to use video visits, we further compared those with and without self-reported access to video-capable technology. The chi-square test was used for comparing categorical variables, and the 2-tailed *t* test was used for comparing continuous variables. We reported all *P* values and considered them to be significant when <.05. Multivariable logistic regression was conducted to identify predictors for willingness to use video visits. All statistical analyses were performed using SAS (version 9.4; SAS Institute, Inc).

### Ethical Considerations

The Miami VA institutional review board granted this study a waiver and deemed it as a quality improvement study (reference number 1360043-3).

## Results

### Survey Respondents

**Figure 1:**
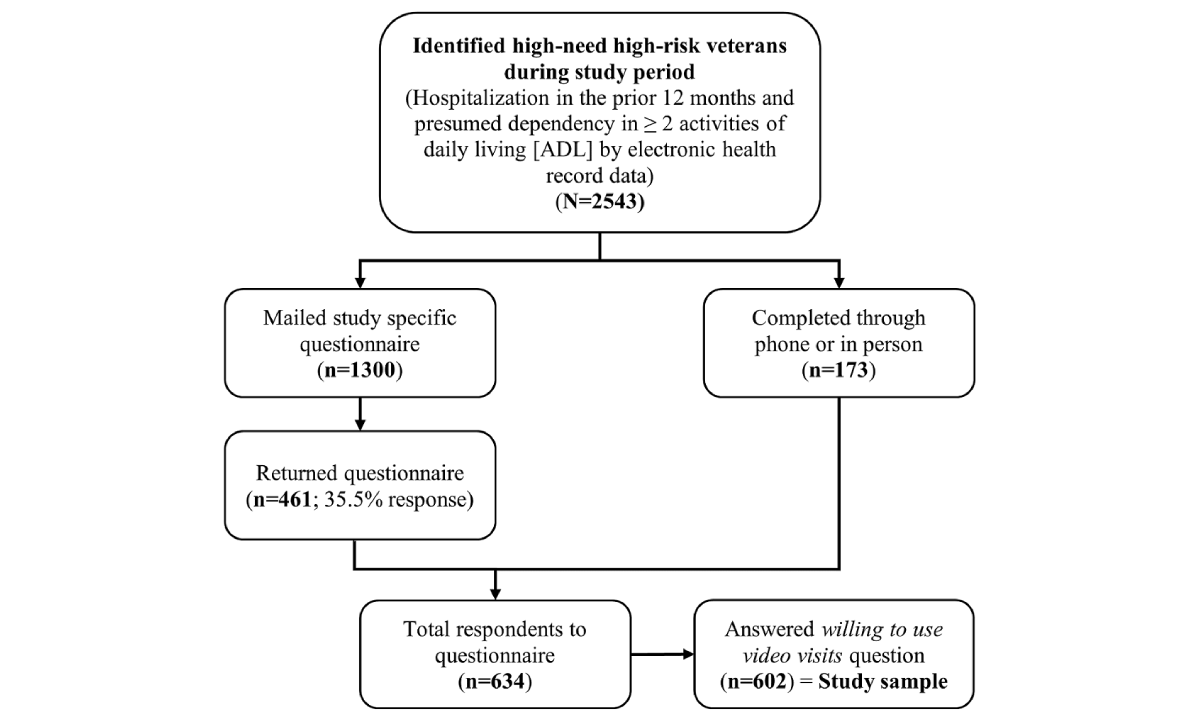
Flowchart showing completed questionnaires.

A total of 1300 HNHR veterans were mailed the questionnaire, of which 461 (35.46%) were returned. In addition, 102 veterans filled the questionnaire over the phone and 71 in person in the frailty clinic, for a total of 634 respondents. Of the 634 individuals returning the survey, 602 (94.9%) respondents answered the *willing to use video visits* question. These 602 respondents represent the main focus of our study (Figure 1). When asked about their willingness to use video visits with their VA care team, 54.3% (327/602) reported their willingness, henceforth labeled as *willing*, whereas 45.7% (275/602) were not willing to use video visits, henceforth labeled as *unwilling*.

The average age of our 602 respondents was 70.6 (SD 9.2; range 39-100) years. Among them, 20.3% (122/602) were aged <65 years, 25.4% (153/602) were aged 65 to 69 years, 25.7% (155/602) were aged 70 to 75 years, 13.8% (83/602) were aged 75 to 79 years, and 14.8% (89/602) were aged >80 years. Only 0.3% (2/602) of the respondents were female. Approximately 61% (367/602) of the respondents were White, 36% (217/602) respondents were African American, and 17.3% (104/602) were Hispanic. Among the 602 responders, 290 (48.2%) were confident filling medical forms by themselves, and 188 (31.2%) had at least an associate degree.

### Difference Between Respondents by Mail versus In Person and Telephone

Individuals completing the survey via mail were significantly more confident filling out medical forms (224/440, 50.9% vs 66/162, 40.7%; *P*=.03); in significantly worse physical health, as measured by their JFI (mean 7.2, SD 1.2 vs mean 6.8, SD 1.2; *P*<.001), Care Assessment Needs scores (mean 94.2, SD 6.8 vs mean 91.6, SD 8.1; *P*<.001), and the total number of Hierarchal Condition Categories conditions (mean 5.7, SD 2.4 vs mean 4.9, SD 1.9; *P*<.001); and significantly more socially isolated (Social Networking Index of 1.5, SD 1.1 vs 1.7, SD 1.1; *P=*.049). There were no other differences between those veterans who completed the mailed survey versus those completing the survey by phone or in person.

Furthermore, we compared the willingness to use video visits between veterans who finished the survey in person and those who did not, and the difference was not significant (*P*=.13). Although more veterans reported no trouble for transportation in those who filled out the survey in person (52/80, 65%) than those who did not (321/531, 60.5%), the difference was not statistically significant (*P*=.05). Similarly, the difference in the percentage of veterans who missed an appointment owing to transportation between those who filled out the survey in person and not in person was not significant (*P*=.28).

### Difference Between Respondents Who Were Willing Versus Unwilling to Use Video Visits

We characterized the differences between 54.3% (327/602) patients *willing* (to use video visits) versus 45.7% (275/602) patients *unwilling* (to use video visits), as shown in Table 2. Those who were willing were significantly younger (average age 68.9, SD 8.8 years) than those unwilling (average age 72.5, SD 9.1 years; *P*<.001). There appears to be a sharp drop in willingness after the age of 75 years.

They were also more likely to have at least an associate educational degree (120/327, 36.7% vs 68/275, 24.7%; *P*=.002) and be more health literate (180/327, 55% vs 110/275, 40%; *P*<.001). Those who were willing were more likely to not use assistive devices for walking (137/327, 41.9% vs 80/275, 29.1%; *P*=.002) and less dependent in their IADL (mean 1.8, SD 2.0 vs mean 2.2, SD 2.2; *P*=.02). Willing veterans reported worse self-rated general health compared with those of unwilling veterans (mean 2.8, SD 0.9 vs mean 3.0, SD 1.0; *P*=.01) and worse physical status (mean 5.2, SD 2.0 vs mean 5.7, SD 2.2; *P*=.004). Willing veterans were also less likely to live in disadvantaged areas (*P*=.048).

When asked about their ability to use technology, a significantly higher number of those willing were able to perform an internet search if given access to a computer (242/327, 74% vs 109/275, 39.6%; *P*<.001); were using email (226/327, 69.1% vs 88/275, 32%; *P*<.001); and were enrolled in the VA’s patient portal, My Health*e*Vet (199/327, 60.9% vs 76/275, 27.6%; *P*<.001). The willing and the unwilling to use video visits groups differed regarding the preferred modes of contact (*P*=.003). Compared with those unwilling to use video visits, willing veterans were more likely to prefer contact by the VA via cell phone (189/327, 57.8% vs 129/275, 46.9%) or via My Health*e*Vet secure message (24/327, 7.3% vs 10/275, 3.6%) and less likely to prefer contact by *landline home phone* (67/327, 20.5% vs 73/275, 26.5%) or mail (44/327, 13.5% vs 57/275, 20.7%).

**Table 2:** Patient characteristics of those willing to use video visits versus not willing to use video visits with their Veterans Affairs care team (N=602).

Characteristics					All completed surveys for study		Willing to use video visits (n=327)		Not willing to use video visits (n=275)		*P* value	
**Demographics**												
	**Age (years)^a^**											
		Values, mean (SD; range)		70.6 (9.2; 39-100)		68.9 (8.8; 39-95)		72.5 (9.1; 42-100)		<.001		
		**Age group, n (%)^a^**										<.001
			<65	122 (20.3)		83 (25.4)		39 (14.2)				
			65-69	153 (25.4)		83 (25.4)		70 (25.5)				
			70-75	155 (25.7)		88 (26.9)		67 (24.4)				
			75-79	83 (13.8)		44 (13.5)		39 (14.2)				
			≥80	89 (14.8)		29 (8.9)		60 (21.8)				
	White, n (%)				367 (61)		207 (63.3)		160 (58.2)		.23	
	African American, n (%)				217 (36)		110 (33.6)		107 (38.9)		.21	
	Hispanic, n (%)				104 (17.3)		56 (17.1)		48 (17.5)		.99	
	Education (at least associate degree)^a^, n (%)				188 (31.2)		120 (36.7)		68 (24.7)		.002	
	Confident filling out medical forms^a^, n (%)				290 (48.2)		180 (55)		110 (40)		<.001	
**Physical health**												
	JEN Frailty Index^a,b^, mean (SD)				7.1 (1.2)		7.0 (1.2)		7.2 (1.2)		.04	
	Care Assessment Needs score^b^, mean (SD)				93.5 (7.3)		93.1 (7.5)		93.9 (7.7)		.20	
	Total number of Hierarchical Condition Categories^b^, mean (SD)				5.5 (2.3)		5.4 (2.3)		5.6 (2.3)		.29	
	FRAIL^b,c^ scale screen positive (score ≥3), n (%)				253 (42)		132 (40.4)		121 (44)		.41	
	Self-rated physical status score^a,d^, mean (SD)				5.4 (2.1)		5.2 (2.0)		5.7 (2.2)		.004	
	Issue with walking, stepping, and balance, n (%)				444 (73.8)		237 (72.5)		207 (75.3)		.49	
	No prosthetic use^a^, n (%)				217 (36)		137 (41.9)		80 (29.1)		.002	
	General health score^a,d^, mean (SD)				2.9 (0.9)		2.8 (0.9)		3.0 (1.0)		.01	
	ADL^e^ score^d^, mean (SD)				84.3 (20.1)		84.9 (19.4)		83.1 (21.0)		.28	
	ADL deficits^b^, mean (SD)				2.3 (2.8)		2.2 (2.6)		2.4 (2.9)		.38	
	IADL^f^ score^a,d^, mean (SD)				6.0 (2.1)		6.2 (2.0)		5.8 (2.2)		.02	
	IADL deficits^a,b^, mean (SD)				2.0 (2.1)		1.8 (2.0)		2.2 (2.2)		.02	
	Homebound or semihomebound, n (%)				169 (28.1)		91 (27.8)		78 (28.4)		.96	
**Mental health**												
	PHQ-2^g^ depression screen positive (score ≥3), n (%)				196 (32.6)		117 (35.8)		79 (28.77)		.08	
	Self-perception of aging score ^b^, mean (SD)				3.2 (1.5)		3.3 (1.5)		3.1 (1.5)		.10	
**Social characteristics**												
	**Area Deprivation Index Score^a,b^, n (%)**											.048
		1-25		113 (18.8)		66 (20.2)		47 (17.1)				
		26-50		155 (25.7)		95 (29.1)		60 (21.8)				
		51-75		192 (31.9)		99 (30.3)		93 (33.8)				
		76-100		138 (22.9)		64 (19.6)		74 (26.9)				
	Have a caregiver, n (%)				204 (33.9)		106 (31)		98 (35.6)		.46	
	Social Networking Index^d^, mean (SD)				1.5 (1.1)		1.6 (1.1)		1.5 (1.1)		.27	
	Having no trouble in transportation, n (%)				373 (62)		198(60.6)		175(63.3)		.49	
	Travel time to physician >60 minutes, n (%)				177 (29.4)		107 (32.7)		70 (25.5)		.06	
	Have delayed physicians’ appointments owing to transportation troubles, n (%)^a^				136 (22.6)		82 (25.1)		54 (19.6)		.14	
**Technology ability^a^—facilitator, n (%)**												
	Use email^a^				314 (52.2)		226 (69.1)		88 (32)		<.001	
	Able to do an internet search^a^				351 (58.3)		242 (74)		109 (39.6)		<.001	
	Use email and internet search				296 (49.2)		214 (65.4)		82 (29.8)		<.001	
	Enrolled in My Health*e*Vet (MHV)^a^				275 (45.7)		199 (60.9)		76 (27.6)		<.001	
	**Preferred mode of contact^a^**											.003
		By home phone		140 (23.3)		67 (20.5)		73 (26.5)				
		By cell phone		318 (52.8)		189 (57.8)		129 (46.3)				
		By MHV secure message		34 (5.6)		24 (7.3)		10 (3.6)				
		By email		101 (16.8)		44 (13.5)		57 (20.7)				

^a^*P*<.05 defined statistical significance.

^b^Lower score is better.

^c^FRAIL: Fatigue, Resistance, Ambulation, Illnesses, and Loss of Weight.

^d^Higher score is better.

^e^ADL: activities of daily living.

^f^IADL: instrumental activities of daily living.

^g^PHQ-2: Patient Health Questionnaire-2.

### Differences Between Willing Respondents With and Those Without Access to Video-Capable Technology

Upon being asked about their access to technology, of the 327 veterans who were willing to use video visits, 248 (75.8%) had a smartphone or computer with a camera, whereas 69 (21.1%) did not. The characteristics of these subgroups are presented in Table 3. Patients with access to the necessary devices were younger (mean 68.3, SD 8.9 vs mean 71.8, SD 8.6; *P*=.004), more health literate (144/248, 58.1% vs 28/69, 41%; *P*=.01), and less likely to be African American (73/248, 29.4% vs 33/69, 48%; *P*=.007) than those without technology access. Veterans with video-capable technology were more functionally independent in their ADL (Barthel ADL score: mean 86.4, SD 17.8 vs mean 77.3, SD 24.3, *P*=.005; number of ADL deficits: mean 2.0, SD 2.5 vs mean 3.2, SD 3.2, *P*=.005) and IADL (Lawton IADL score 6.3, SD 1.9 vs 5.7, SD 2.2, *P*=.04; and number of IADL deficits 1.7, SD 1.9 vs 2.3, SD 2.2 and *P*=.04). They were less likely to report issues with walking, stepping, or balance (173/248, 69.8% vs 58/69, 84%; *P*=.03) and more likely to not use assistive devices for walking (115/248, 46.4% vs 18/69, 26%; *P*=.004). They were less likely to live in disadvantaged areas (*P*=.049). They were also less likely to have trouble with transportation (167/248, 67.3% vs 25/69, 36%; *P*<.001) and less likely to have delayed their physicians’ appointments because of transportation troubles (54/248, 21.8% vs 26/69, 38%; *P*=.01). Veterans with access to a video-capable device were more likely to be able to use the internet (204/248, 82.3% vs 28/69, 41%; *P*<.001), use email (196/248, 79% vs 20/69, 29%; *P*<.001), and be enrolled in My Health*e*Vet (173/248, 69.8% vs 17/69, 25%; *P*<.001).

**Table 3:** Patient characteristics by access to a video-capable technology of those willing to use video visits who answered both questions (N=317).

Characteristics					Access to a video-capable device (n=248)		No access to a video-capable device (n=69)		*P* value	
**Demographics**										
	**Age (years)**									
		Values, mean (SD; range)			68.3 (8.9; 39-95)		71.8 (8.6; 55-94)		.004	
		**Age group, n (%)^a^**								.02
			≤64	68 (27.4)		12 (17.4)				
			65-69	64 (25.8)		14 (20)				
			70-74	66 (26.6)		21 (30)				
			75-79	31 (12.5)		12 (17)				
			≥80	19 (7.7)		10 (14)				
	White, n (%)^a^				168 (68)		34 (49)		.007	
	African American, n (%)^a^				73 (29.4)		33 (48)		.007	
	Hispanic, n (%)				46 (18.5)		9 (13)		.37	
	Education (at least associate degree), n (%)				91 (36.7)		22 (32)		.55	
	Confident filling out medical forms, n (%)^a^				144 (58.1)		28 (41)		.01	
**Physical health**										
	JEN Frailty Index^b^, mean (SD)				7.0 (1.1)		7.1 (1.2)		.53	
	Care Assessment Needs score^b^, mean (SD)				92.9 (7.0)		93.4 (6.7)		.59	
	Total number of Hierarchical Condition Categories^b^, mean (SD)				5.4 (2.5)		5.5 (1.9)		.72	
	FRAIL^c^ scale screen positive (score ≥3), n (%)				100 (40.3)		28 (41)		.99	
	Physical status score^d^, mean (SD)				5.3 (2.0)		5.0 (1.8)		.23	
	Issue with walking, stepping, balance, n (%)^a^				173 (69.8)		58 (84)		.03	
	No prosthetic use, n (%)^a^				115 (46.4)		18 (26)		.004	
	General health score^a,d^, mean (SD)				2.8 (0.9)		2.6 (0.8)		.08	
	ADL^e^ score^a,d^, mean (SD)				86.4 (17.8)		77.3 (24.3)		.005	
	ADL deficits^a,b^, mean (SD)				2.0 (2.5)		3.2 (3.2)		.005	
	IADL^f^ score^a,d^, mean (SD)				6.3 (1.9)		5.7 (2.2)		.04	
	IADL deficits^a,b^, mean (SD)				1.7 (1.9)		2.3 (2.2)		.04	
	Homebound or semihomebound, n (%)				70 (28.2)		17 (25)		.66	
**Mental health**										
	PHQ-2^g^ screen positive (score ≥3), n (%)				86 (34.7)		26 (38)		.75	
	Self-perception of aging score^b^, mean (SD)				3.2 (1.6)		3.5 (1.3)		.11	
**Social characteristics**										
	Area Deprivation Index Score^a,b^, n (%)									.49
		1-25			54 (21.8)		11 (15.9)			
		26-50			76 (30.6)		19 (28)			
		51-75			69 (27.8)		25 (36)			
		76-100			46 (18.5)		14 (20)			
	Have a caregiver, n (%)				81 (32.7)		25 (36)		.68	
	Social Networking Index^d^, mean (SD)				1.6 (1.1)		1.5 (1.1)		.51	
	Have no trouble with transportation, n (%)^a^				167 (67.3)		25 (36)		<.001	
	Travel time to physician >60 minutes, n (%)				83 (33.5)		23 (33)		.99	
	Have delayed physicians’ appointments owing to transportation troubles, n (%)^a^				54 (21.8)		26 (38)		.01	
**Technology ability, n (%)^a^**										
	Use of email^a^				196 (79.0)		20 (29)		<.001	
	Able to do an internet search^a^				204 (82.3)		28 (41)		<.001	
	Use email and internet search				187 (75.4)		17 (25)		<.001	
	Enrolled in My Health*e*Vet (MHV)^a^				173 (69.8)		17 (25)		<.001	
	**Preferred mode of contact^a^**									.03
		By home phone			46 (18.6)		20 (29)			
		By cell phone			144 (58.1)		39 (57)			
		By MHV secure message			22 (8.9)		0 (0)			
		By email			34 (13.7)		9 (13)			

^a^*P*<.05 defined statistical significance.

^b^Lower score is better.

^c^FRAIL: Fatigue, Resistance, Ambulation, Illnesses, and Loss of Weight.

^d^Higher score is better.

^e^ADL: activities of daily living.

^f^IADL: instrumental activities of daily living.

^g^PHQ-2: Patient Health Questionnaire-2.

### Number of Willing Respondents With Access and Ability to Use Video Visits

In our HNHR group, 54.3% (327/602) were willing to receive care from their VA health care team via video visits (Table 2), and of those, 78.2% (248/317) had access to video-capable technology (Table 3). Therefore, 41.2% (248/602) participants were willing and had the technology for a video visit. Among the willing 248 patients with access to a video-capable device, only 204 (82.3%) were likely to be comfortable using technology when factoring in previous use of the internet or email (Table 3). Therefore, the percentage of HNHR veterans with access and ability likely decreases to approximately 33.9% (204/602).

### Multivariable Logistic Regression

Multivariable logistic regression was conducted to give a sense of the relative importance of different predictors of willingness. The odds ratios for willingness estimated for age, degree, confidence in filling out forms, JFI score, self-perception of health, prosthetics use, general health, IADL score, ADI, use of email, use of the internet, and My Health*e*Vet use are presented in Table 4. As shown in Table 4, age, confidence in filling out forms, prosthetics use, general health, and use of the internet were significantly associated with willingness of video visit use in the multivariable analysis, indicating that they are the strongest predictors compared with others that were only significant in the univariate analysis.

**Table 4:** Odds ratio for predictors of willingness to use video visits in multivariable logistic regression.

Characteristics	Odds ratio (95% CI)	*P* value
Age	0.97 (0.95-0.995)	.02
Education (at least associate degree)	1.39 (0.94-2.07)	.10
Confidence in filling medical forms	1.47 (1.01-2.14)	.046
JEN Frailty Index score	1.03 (0.89-1.20)	.70
Self-perception of aging	0.92 (0.82-1.03)	.15
Prosthetics use	1.85 (1.23-2.80)	.003
General health	0.72 (0.56-0.92)	.01
Instrumental activities of daily living score	1.03 (0.93-1.14)	.60
Area Deprivation Index score	0.99 (0.99-1.00)	.06
Use of email	0.98 (0.67-1.43)	.91
Use of the internet	2.34 (1.65-3.34)	<.001
My Health*e*Vet use	1.29 (0.90-1.85)	.17

## Discussion

### Principal Findings

Our study aimed to identify the readiness of using video visits for health care by assessing willingness, access, and ability in older HNHR patients with complex needs, functional limitations, and a variety of chronic conditions [30]. A little over half were willing to use video visits, three quarters of those had access, and only 80% of them were comfortable with technology. Overall, we believe that only one-third of the HNHR veterans had the willingness, access, and ability to use video visits for health care. Therefore, data from our project suggest that among vulnerable HNHR older adults, the proportion not ready for video visits may be much higher than the one-third previously reported for a cross-section of community-dwelling older adults [9] and likely is approximately two-thirds of the HNHR veterans.

The access gap between those willing yet without technology was larger among those who were older, less health literate, or African American or lived in disadvantaged areas. Veterans who did not have a device were less healthy, more likely to be dependent and have transportation challenges, and less well-versed with using the internet and email. In contrast, veterans who were willing to use video visits were younger, more literate, more adept at using technology, more functionally independent in their IADL, and less likely to live in disadvantaged areas but had worse self-rated health. Age, confidence in filling out forms, prosthetic use, general health, and internet use were significantly associated with willingness to use video visits in the multivariable analysis. Age is a strong predictor, and there appears to be a sharp drop in willingness after the age of 75 years. Moreover, there was a very strong correlation of both technology access and digital skills on willingness.

In addition, willingness was correlated with a previous history of having missed their in person physicians’ appointments because of issues involving transportation. Although the HNHR population’s willingness to use video visits represents an opportunity to address critical access barriers often seen in this population, these inequities in access to video visits and their lack of prior technology use warrant further attention, as reliance on telemedicine visits could exacerbate the gap in access to care for vulnerable populations. Although there were no differences in the willingness to use video visits and insignificant differences in transportation barriers between veterans who finished the survey in person and those who did not, individuals completing the survey via mail were more confident filling out medical forms but in worse physical health and more socially isolated than those who completed by phone or in person. Although not significant, these results may be an indirect reflection regarding the availability of resources for attending in person appointments and need further inspection.

Owing to the unprecedented challenges to health care during the COVID-19 pandemic, there has been a substantial increase in patients’ willingness to use technology to reduce in person appointments to safeguard against COVID-19 [2]. However, even as telemedicine willingness increases, not only is it necessary to address the lack of access to technology in and of itself but also other strategies to address telemedicine unreadiness are needed. Some ways of addressing technology access challenges may be providing necessary equipment and bandwidth via the health care system [31] or helping patients acquire affordable devices and broadband internet [11]. In August 2020, the Assistant Under Secretary for Health for Clinical Services submitted a memorandum for expanding access to telehealth for veterans through a digital divide consult. This consult is available to veterans who do not have a video-capable device or connectivity for eligibility in participating in the Lifeline program to receive a loaned device (eg, iPads or iPhones) for accessing telemedicine in their home or location of choice. The VA offers tablets and data plans to veterans who qualify using a digital divide consult and has simplified the use of technology for video visits by configuring VA-loaned tablets to allow for a single-use mode [31]. The single-use mode replaces the complexity of multiple VA functions, features, and apps on the device with a VVC icon that readily connects the veteran to a telemedicine medical room [31].

Strategies are needed to address technology literacy and offer necessary education and support so that patients may engage successfully in video visits. Specific outreach efforts need to target communities that have been found to be less ready for video visits, including African Americans and those with high area deprivation scores. More systems need to implement initiatives that enable trained staff or even volunteers to help patients navigate the complexities of devices and applications [4] and programs that enhance self-efficacy, which have proven successful in the adoption of technology [32]. Other potential approaches include offering technology education and support, using nonmedical staff to conduct a mock visit before the actual visit to train older adults in navigating the technology, using trained peers or community health workers to provide in-home training or act as telepresenters for in-home video visits with high-risk older adults, and encouraging family caregivers and friends to participate during telemedicine encounters.

Moreover, the presentation of video versus in person visits is somewhat of a false dichotomy. Video visits may have more capacity to address multi-morbid diseases, as indicated by longer visit durations and a larger number of visit diagnoses than those of telephone visits [33]. However, there is a population that has significant barriers to both physical (transportation) and video (digital literacy) interactions. For this group, telephone visits may be more accessible than video or in person visits and can potentially be another means of increasing care. For a few patients, neither telemedicine (telephone or video) nor in person may be feasible, and home care models such as Medicare’s Independence At Home and VA’s HBPC may be necessary.

This study has several strengths. A strength of our study is that it specifically assesses an older, functionally dependent, HNHR population with complex needs and social isolation. We used a novel VA set of HNHR older adults and surveyed them about their attitudes toward telemedicine and their physical, emotional, and social determinants. In addition to characterizing the willingness, access, and ability to use video visits for health care, in the context of their physical, emotional, and social characteristics, as has previously been done [9,11-13], we correlated it to frailty status and the neighborhood they reside in.

However, this study does have several limitations. One of the limitations is that technology access was only asked for those who were willing. Had we surveyed our total study sample regarding access, the proportion of those lacking access would likely be higher, given the lower use of email and internet and lower education level and health literacy among the unwilling veterans. Moreover, we did not explore the reasons driving the unwillingness to use video visits, explore the subgroup that has the technology but is unwilling, or include an *uncertain* response category for willingness in our survey. Understanding their barriers and facilitators might provide important insights beyond affordable access to devices and connectivity and digital skills [34]. Previous reports suggest that in addition to poor technology access and literacy, technology unwillingness may be driven by several other factors, including sensory or memory impairment [9,12], which we did not assess. The ADI does not explicitly incorporate neighborhood availability of affordable broadband, which may be a big factor in whether or not people use it; however, it may reflect digital redlining [34].

We also did not ask about or compare willingness among those who had versus did not have prior telehealth visits. Some of the constructs are somewhat narrowly assessed: specifically, the social support measure that assesses caregiver presence with an unvalidated question. However, this was supplemented by the Berkman–Syme Social Network Index, which takes into account marital status, frequency of meeting and talking to close friends and relatives, and participating in religious and club meetings. Similarly, mental health is assessed with a validated 2-item depression scale and is therefore supplemented by the 5-item Self-Perception of Aging scale. Another limitation is that our population was US veterans and overwhelmingly male. The gender demographics here reflect that of the VA, where 89.6% of all veterans are male [35], and not of the general older adult population. Older female HNHR patients may have different needs and access challenges than those described in this study. Moreover, our study was urban and limited geographically to the Miami area and, thus, may not represent regional variations. In addition, we did not assess the availability of the caregivers who may be willing and able to help with the video visit and may have access to the needed devices. Adjustment for multiple comparisons tends to increase type II error [36,37]; therefore, we did not adjust for multiple comparisons. Other limitations include a relatively low survey response rate. The survey was also conducted for patients in an integrated health care system, which may make the findings less generalizable to patients from other types of systems.

### Conclusions

Our results underscore the well-recognized fact that older adults, a group that uses health care at one of the highest rates, face significant barriers to accessing needed care, whether it be in person or telemedicine. Certain characteristics put individuals within this group at an even higher risk for barriers to care. Future research is needed to urgently explore ways of mitigating the identified obstacles to telemedicine among HNHR patients at a system level and study and address potential barriers such as concerns about care quality and relationships with physicians at the patient–provider level [38,39]. Programs for HNHR patients should address the specific factors identified here to pave the way for equitable access to health care among high-risk patients. It is recognized that individuals’ characteristics, as well as the surrounding social and health care system, are the most important factors that affect telemedicine adoption [40], and some may also serve as barriers. Thus, it was difficult to make a distinction. However, it is important to recognize that only some of the factors are modifiable; thus, the need to make a distinction may be less pertinent. These respondents completed the survey before the COVID-19 pandemic, and it is possible that the COVID-19 pandemic may have significantly changed patients’ video acceptance and technology availability as they may have adopted video for personal and health reasons [2]. Thus, the development of innovative, sustainable strategies to support and improve care access for this vulnerable population will help during the COVID-19 pandemic; however, it will also help better manage HNHR patients and keep them healthy in their homes for as long as possible after the COVID-19 pandemic.

## Abbreviations

ADI: Area Deprivation Index
ADL: activities of daily living
CMS: Centers for Medicare and Medicaid Services
HBPC: home-based primary care
HNHR: high-need, high-risk
IADL: instrumental activities of daily living
JFI: JEN Frailty Index
VA: Veterans Affairs
VVC: Veteran Video Connect
